# White Matter Hyperintensity in Patients with Sudden Sensorineural Hearing Loss †

**DOI:** 10.3390/diagnostics14111109

**Published:** 2024-05-27

**Authors:** Mehdi Abouzari, Arash Abiri, Karen Tawk, Cynthia Tsang, Beenish Patel, Avissa Khoshsar, Hamid R. Djalilian

**Affiliations:** 1 Department of Otolaryngology–Head and Neck Surgery, University of California, Irvine, CA 92697, USA; 2 Department of Biomedical Engineering, University of California, Irvine, CA 92697, USA; 3 Department of Neurological Surgery, University of California, Irvine, CA 92697, USA

**Keywords:** sudden sensorineural hearing loss, magnetic resonance imaging, white matter hyperintensity, Fazekas scale, Mirsen scale

## Abstract

Objective: To compare white matter hyperintensities (WMHs) on T2-weighted magnetic resonance imaging (MRI) of patients with sudden sensorineural hearing loss (SSNHL) and analyze subpopulations with age-matched controls. Methods: T2-weighted MRI scans of 150 patients with SSNHL were assessed for WMHs and compared with the data of 148 healthy age-matched adults. Assessments of WMHs included independent grading of deep white matter hyperintensities (DWMHs) and periventricular hyperintensities (PVHs). WMH severity was visually rated using the Fazekas and Mirsen scales by two independent observers. Results: Fazekas grades for PVHs (*p* < 0.001) and DWMHs (*p* < 0.001) of SSNHL patients were found to be significantly greater than those of healthy participants. The average Mirsen grades for DWMHs of healthy and SSNHL patients were evaluated to be 0.373 ± 0.550 and 2.140 ± 0.859, respectively. Mirsen grades for DWMHs of SSNHL patients were found to be significantly greater (*p* < 0.001) than those of healthy participants. The Mirsen scale was found to have higher sensitivity (*p* < 0.001) than the Fazekas scale in grading PVHs and DWMHs. No significant difference (*p* = 0.24) was found in specificities between the two scales. Conclusions: Patients with sudden hearing loss have a much higher likelihood of having periventricular and deep white matter hyperintensities compared to age-matched controls. These findings indicate that sudden hearing loss patients are more likely to have microvascular changes in the brain, which may indicate a vascular and/or migraine origin to sudden sensorineural hearing loss.

## 1. Introduction

Sudden sensorineural hearing loss (SSNHL) is defined as a ≥30 dB reduction in the sensorineural hearing threshold at three consecutive frequencies, occurring rapidly within a 72 h period [1,2]. The estimated annual incidence of SSNHL ranges from 5 to 27 per 100,000 people, amounting to approximately 66,000 new cases per year in the United States [3,4]. Patients often present after waking up with hearing loss, while others describe their hearing loss as being preceded by a “pop” sound. SSNHL affects men and women equally, and nearly all cases are unilateral. A limited understanding of the underlying pathophysiology and causative entities (e.g., idiopathic, vascular, viral, infections, autoimmune, membrane rupture, etc.) [5] has led to ongoing discussions regarding the definitive diagnosis and treatment of SSNHL [6,7]. A plethora of therapeutic avenues exist for these patients, though most receive oral or intratympanic steroids. Other treatment strategies include antiherpetic therapy, diuretics, hyperbaric oxygen therapy, and fibrinogen/LDL-apheresis, among others [2,8,9]. However, there is no consensus on efficacious treatment options. While some research suggests that one- to two-thirds of patients may recover hearing spontaneously, several factors indicate less favorable outcomes. These include more severe initial hearing loss, delayed or no steroid treatment, and a history of hearing loss or vestibular disorders [10,11]. Pure tone audiometry remains the mainstay for assessing idiopathic SSNHL and also provides prognosis value. Additionally, imaging tests such as magnetic resonance imaging (MRI) can help detect hearing loss-inducing lesions [6,8]. The American Academy of Otolaryngology–Head and Neck Surgery Foundation’s clinical practice guideline on sudden hearing loss strongly recommends obtaining an MRI to rule out retrocochlear pathology, despite most cases having unidentifiable causes [2]. In addition, several studies have suggested that various MRI sequences can reveal SSNHL diagnostic signs such as inflammation, blood–labyrinthine barrier breakdown, hemorrhage, or microangiopathic brain changes [12,13,14,15,16].

An emerging theoretical etiology of idiopathic SSNHL includes the vascular insult to the cochlea [8,16], which aligns with reported associations between SNHL and other vascular pathologies such as cardiovascular diseases [17,18] or migraine [19,20,21,22]. Hilger was among the first to describe the autonomic dysfunction of the inner ear, emphasizing the delicate nature of the inner ear vasculature, which is supplied by end-arteries. He described how minute vascular changes can result in sensory disturbances such as hearing loss, depending on the branch involved [23]. In addition, epidemiological evidence suggests a strong correlation between SSNHL and cerebrovascular disorders, as evidenced by a study with over 40,000 participants indicating that stroke patients have a 71% increased risk of developing SSNHL [24]. Other reports using MRI have documented heightened arterial stiffness and a greater prevalence of leukoaraiosis, which are deep white matter abnormalities observed in CT or MRI brain scans, in patients with SSNHL. This supports the theory that vascular factors play a role in this condition [25]. Interestingly, previous studies have found correlations between migraine diagnosis and white matter abnormalities on MRI, and the severity of white matter hyperintensities (WMHs) has also been correlated with migraine symptom severity and prognosis [26,27,28]. In migraineurs, WMHs are found in different locations (deep or subcortical) compared to the general population (periventricular) and are noticed earlier in age [29,30,31].

Several studies are now drawing connections between leukoaraiosis and small vessel disease, lacunar infarction, hypertension, dementia, and death, challenging the previous notion that these were “age-related spots” in the brain [32,33,34]. Eckert and colleagues have demonstrated that MRI WMHs may be a reliable marker for cerebral small vessel diseases [35] and age-related low-frequency hearing loss [36]. Furthermore, a recent case-controlled study by Dicuonzo et al. demonstrated a high prevalence of WMHs among patients with SSNHL [37]. The investigators looked at 36 subjects diagnosed with unilateral SSNHL alongside age- and sex-matched controls, finding significantly higher WMH values in patients with SSNHL than in the control group. While occasionally benign, WMHs have been shown to be suggestive of an underlying cardiovascular disease or neurological condition, such as cerebrovascular disease or stroke, cognitive decline, or dementia [34]. WMHs are usually classified into either periventricular hyperintensities (PVH) or deep white matter hyperintensities (DWMH). PVHs have traditionally been associated with aging (e.g., cerebral hypotension, hypoperfusion, and atrophy), while DWMHs have been associated with atherosclerosis and endothelial inflammation [38]. Differences between the two lesions can be explained pathologically, with both presenting with demyelination, gliosis, and fiber loss. However, PVHs were shown to worsen as fiber loss worsened, while DWMHs worsened with tissue loss. Several studies have indicated that tissue and vascular changes spread further than the visible sites of WMH [39].

The Fazekas scale and the Mirsen scale are radiological tools used to evaluate these white matter changes in the brain [40]. The Fazekas scale assesses the severity of white matter changes on a 4-point scale (ranging from 0 to 3) based on the presence and extent of PVH and DWMH lesions. On the other hand, the Mirsen scale evaluates WMHs based on three categories (periventricular, deep, and infratentorial) and scores each on a 5-point scale (ranging from 0 to 4) based on the number and size of WMHs present. While both scales have been used to provide standardized and objective evaluation of white matter changes, they have not been directly compared for differences in sensitivities or clinical relevance. In this manuscript, we aim to utilize the Fazekas and Mirsen scales to evaluate the prevalence of MRI WMHs in patients with SSNHL compared to healthy controls and assess the specificity and sensitivity of these radiologic scales in SSNHL.

## 2. Methods

### 2.1. Patient Selection and Data Collection

With the Institutional Review Board (IRB) approval, a publicly available dataset (https://www.humanconnectome.org/study/hcp-young-adult, accessed on 8 April 2024) from the Human Connectome Project (HCP) was used to obtain the data of 148 healthy (no significant history of psychiatric disorder, substance abuse, neurological, or cardiovascular disease) adults with an age range of 22–35 years old [41]. All experimental procedures were performed under HCP guidelines. Additionally, this study retrospectively reviewed data from 150 subjects who presented to our tertiary care otology and neurotology clinic and were subsequently diagnosed and treated for SSNHL. The T2-weighted MRI sequence of each subject was obtained and analyzed under IRB approval. The MRIs from SSNHL subjects were scanned with the same equipment and protocol. The control group had been scanned with similar equipment at a different institution.

### 2.2. Assessment of White Matter Hyperintensities

**Figure 1:**
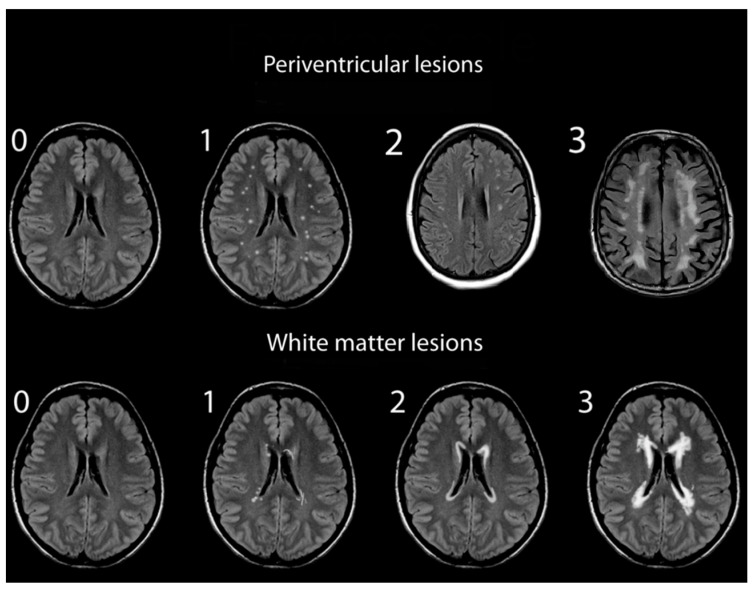
Grading of periventricular and deep white matter hyperintensities based on Fazekas scale.

**Figure 2:**
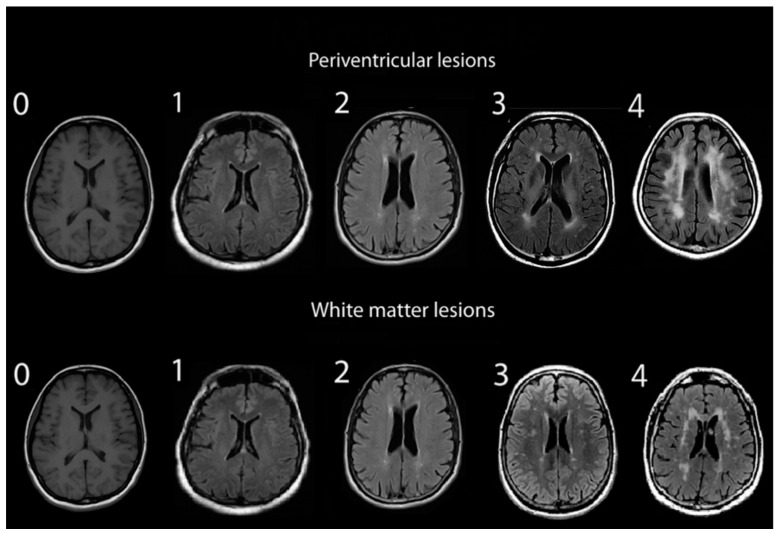
Grading of periventricular and deep white matter hyperintensities based on Mirsen scale.

Assessments of WMHs included independent grading of DWMHs and PVHs. All WMHs were evaluated by a single rater blinded to the clinical data of study participants, using only the axial views of subject MRIs. Infratentorial hyperintensities and basal ganglia were not rated as part of this study. WMH severity was visually rated using the Fazekas and Mirsen scales [42,43]. The Fazekas scale graded WMHs as 0—absent; 1—punctuate; 2—early-confluent; and 3—confluent (Figure 1). DWMHs and PVHs with Fazekas grades greater than 1 were classified as progressive, whereas those with grades 0 to 1 were considered benign [44,45]. The Mirsen scale graded the number of DWMHs as 0—absent; 1—one or two focal lesions; 2—three to five lesions; 3—more than five lesions; 4—confluent lesions (Figure 2). DWMHs with Mirsen grades greater than 1 were considered progressive while grade 0 corresponded to absent WMH. Likewise, the Mirsen scale graded PVHs as either absent (benign) or present (progressive).

### 2.3. Statistical Analyses

Continuous variables were described as the mean ± standard deviation. All statistical calculations were performed in MATLAB (MathWorks Inc., Natick, MA, USA). Wilcoxon rank sum tests were utilized to assess statistical differences between means. A Fisher Exact test was used to evaluate differences between two categorical variables. A *p*-value of <0.05 was considered statistically significant.

## 3. Results

Among the healthy patient cohort, 62 (42%) were male and 87 (58%) were female; exact patient ages were not available, though all were between 22 and 35 years old. SSNHL patients were 57% male and 43% female, with a mean age of 55 ± 17 years. Mean Fazekas and Mirsen grades of PVHs and DWMHs among healthy and SSNHL patients were evaluated (Table 1). The average Fazekas grades for PVHs of healthy and SSNHL patients were 0.020 ± 0.141 and 1.273 ± 0.684, respectively (*p* < 0.001). Assessments for DWMHs of these two groups using the Fazekas scale yielded average grades of 0.041 ± 0.198 and 1.073 ± 0.743, respectively (*p* < 0.001). The average Mirsen grades for DWMHs of healthy and SSNHL patients were evaluated to be 0.372 ± 0.551 and 2.140 ± 0.859, respectively (*p* < 0.001). Mirsen grading demonstrated the presence of PVH in 95% of the SSNHL MRIs and 2% of the healthy MRIs (*p* < 0.001).

To minimize the confounding effect of age, we considered a subpopulation of 25 age- and sex-matched patients with SSNHL to compare with the healthy controls. All patients were between 18 and 45 years old (no significant difference in mean age compared to the healthy cohort) and 14 (56%) patients were female (*p* = 0.77). Of the 25 patients with SSNHL, 24 (96%) had PVH based on either Mirsen or Fazekas scales, while only 3 out of 148 healthy controls (2%) had PVH (*p* < 0.001). In addition, 25 out of the 25 patients with SSNHL (100%) had WMH, whereas 51 out of 148 healthy controls (34%) had WMH based on the Mirsen scale (*p* < 0.001). The Fazekas scale showed 19 out of 25 patients (76%) and 6 out of 148 healthy controls (4%) with WMH (*p* < 0.001) (Table 2).

**Figure 3:**
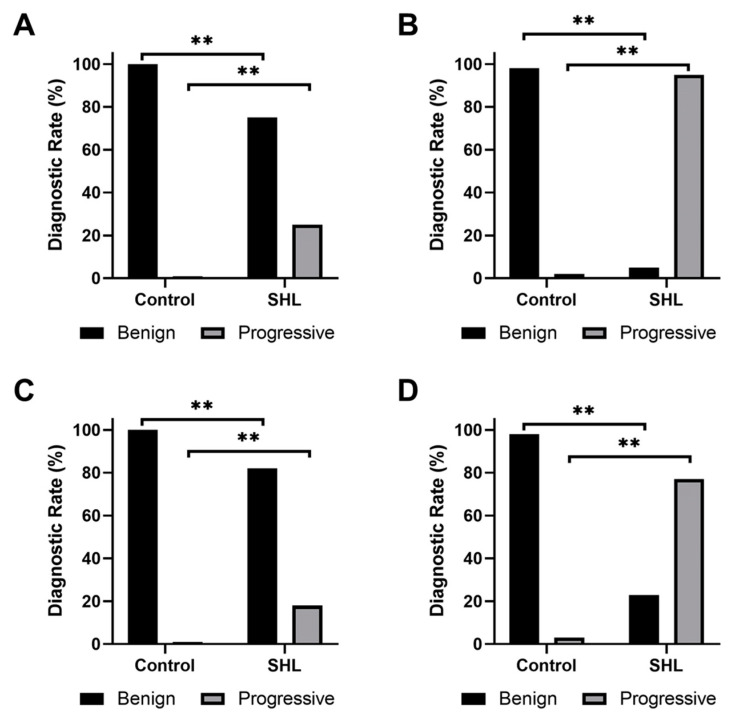
Bar plots of the frequency of (**A**,**B**) periventricular hyperintensities and (**C**,**D**) deep white matter hyperintensities, classified as benign or progressive according to (**A**,**C**) Fazekas or (**B**,**D**) Mirsen scale. ** indicates a *p* < 0.001.

Fazekas and Mirsen scales were used to classify DWMHs and PVHs as either benign or progressive (Figure 3). A significantly larger number of WMHs in SSNHL subjects were found to be progressive compared to healthy participants (*p* < 0.001). Sensitivities and specificities of Fazekas and Mirsen scales in grading PVHs and DWMHs for diagnosis of patients with SSNHL were calculated (Table 3). The Mirsen scale was found to have higher sensitivity than the Fazekas scale in grading PVHs (*p* < 0.001). No significant difference (*p* = 0.248) was found in specificities between the two scales. Similarly, the Mirsen scale was determined to have higher sensitivity in grading DWMHs compared to the Fazekas scale (*p* < 0.001). However, these two scales demonstrated no significant difference in their specificities (*p* = 0.060). While there was no difference in specificities (*p* = 0.723), the use of the Mirsen scale for assessing PVHs demonstrated significantly higher sensitivity (*p* < 0.001) in SHL diagnosis than evaluating Mirsen grades for DWMHs.

## 4. Discussion

This manuscript compared MRIs of patients with idiopathic SSNHL to healthy individuals and demonstrated significantly higher WMH severities according to Fazekas and Mirsen gradings in SSNHL patients. We observed that patients with SSNHL had a significantly higher number of progressive DWMH and PVH compared to healthy controls. Furthermore, though Mirsen grading was more sensitive in detecting PVH and DWMH compared to Fazekas grading, both scaling systems were as equally specific in detecting WMH in patients with SSNHL. These findings along with related reports from the literature support a possible vascular etiology as part of the underlying pathophysiology causing idiopathic SSNHL.

Comprehensive reviews by Chau et al. and Kuhn et al. have suggested a possible association between idiopathic SSNHL and vascular and hematologic pathologies [5,8]. This vascular theory attributes SSNHL symptoms to damage, ischemia, hemorrhage, or oxygen deprivation of terminal arteries supplying the cochlea, thus damaging spiral ganglion or hair cells and causing hearing loss [36,46,47]. Several studies have reported imaging evidence of labyrinthine hemorrhage in SSNHL [48,49]. Additionally, vascular-specific characteristics such as imbalanced homocysteine, folate, adhesion molecules, oxidative agents, and circulating endothelial progenitor cell levels have been denoted in SSNHL [37]. In line with the vascular theme, a higher prevalence of migraine diagnosis, which is a complex neurovascular disorder, has been observed in patients with SSNHL [19,22,50]. A population-based study examining more than ten thousand migraine patients concluded that migraine was a risk factor for developing idiopathic SSNHL [20].

The utilization of WMH on T2-weighted MRI to elucidate cerebral small vessel diseases has been previously demonstrated in the literature [26,35]. A meta-analysis by Swartz et al. demonstrated that MRI white matter abnormalities were also associated with migraine [27]. Likewise, recent institutional studies have demonstrated a significant prevalence of WMH among migraine where the WMH severity could be associated with worse symptoms and prognosis [28,29]. In contrast to the typical age-related WMH observed in the general population, migraine has been linked to WMHs located in the deep or subcortical areas rather than the periventricular areas [30,31]. It is important to note that cardiovascular risk factors were not found to be more common among migraineurs with WMH, and these WMHs tend to occur earlier in the lives of migraineurs [51]. These findings might explain the increased frequency of SSNHL within the migraine population. WMHs, which are believed to be caused by multiple microemboli [52], might induce SSNHL when affecting the inner ear circulation. Despite these insights, the studies investigating WMH’s clinical significance in the context of migraine and SSNHL remain scarce.

In a novel study assessing WMHs in low-frequency hearing loss, particularly in women with high blood pressure, Eckert and colleagues demonstrated an association between hearing loss and small vessel disease evidenced by MRI WMH [36]. In addition, Shin et al. reported higher Fazekas scale scores among patients with idiopathic SSNHL, who also had diabetes mellitus and/or hypertension. In addition, they found better baseline hearing thresholds in the affected side, and lower Fazekas scale scores were correlated with complete treatment response [53]. Furthermore, two Italian studies investigated the correlation between SSNHL and WMH. The first study revealed that individuals with SSNHL, aged between 48 and 60 years, had a 26% higher likelihood of obtaining a score of 1 on the Fazekas scale compared to the control group [54]. The second group reported that an increased Fazekas scale score corresponded with a lower probability of hearing recovery, decreasing from 71% for those with a score of 0 to 15% for those with scores of 3 and 4 [55]. Our study demonstrated distinct WMH differences between patients with SSNHL and healthy controls, further supporting the microvascular theory as the etiology of idiopathic SSNHL.

Prior to this study, most articles investigating the utility of MRI in the SSNHL patient population have focused on detecting neoplastic lesions such as vestibular schwannomas [56,57] or three-dimensional fluid-attenuated inversion recovery sequencings’ demonstration of cochlear signals and inner-ear fluid spaces [13,58]. On the other hand, our understanding of WMH was mostly limited to its association with aging until recently [37,59]. This study is among the first to examine WMHs as a possible marker for idiopathic SSNHL, demonstrating greater MRI DWMH and PVH profiles compared to healthy controls. By performing WMH analysis via two different grading scales and observing significant differences between SSNHL and controls as well as a high sensitivity and specificity, this study supports the vascular theory of SSNHL. This study also gives further credence to the associations between SSNHL and other vascular etiologies such as migraines [21,22]. Similar to the current study, a recent paper by Dicuonzo et al. observed a high prevalence of WMHs in MRIs of patients with SSNHL patients compared to controls [37]. In addition, they noted a higher recovery rate in patients with greater PVHs, which contrasts with the findings of the Italian studies mentioned earlier. Another study identified a protein kinase gene associated with the risk of cerebral infarction and hemorrhage that also increased the risk for SSNHL in patients who had higher WMH [60]. While we found a strong association between SSNHL and WMH, it should be noted that the vascular (likely migraine) theory is not the sole cause of SSNHL but may be the etiology in the majority of patients. For instance, Hiramatsu and colleagues explored the relationship between polymorphisms in inflammatory mediator genes and SSNHL susceptibility. Their hypothesis is based on the theory that inflammation contributes to increased blood vessel permeability, potentially leading to WMH. Their findings supported this hypothesis, indicating a connection between inflammation, which often results in increased vascular permeability in the inner ear of patients with SSNHL, and the occurrence of this condition [61]. Future research is warranted to investigate this phenomenon thoroughly and assess the efficacy of novel treatment options.

Despite our efforts to appropriately analyze and interpret the data, it is important to note several limitations of this study. First, MRIs of the experimental and healthy patients were derived from different patient populations and were not matched based on demographic and clinical characteristics such as past medical histories, family histories, and other comorbidities. It has been shown that age, vascular risk factors, and inflammation-related genetic variants may have an impact on the presence of WMHs in healthy controls even in the absence of comorbidities [62]. Several studies have reported the presence of WMHs in the younger population, ranging from 5.3% to 50.9% [63,64,65,66]. For example, in a study of 1249 healthy patients aged 1–45 years with no history of stroke, traumatic brain injury, neoplasm, psychiatric illness, demyelinating disease, metabolic disease, or substance abuse, 25.9% of subjects were found to have WMHs on MRI [66]. This is important to consider for future studies since other vascular comorbidities such as stroke and cognitive decline have been associated with WMH [56,59,67]. However, other confounders such as age and sex were adjusted by comparing the age- and sex-matched SSNHL patients to healthy controls, thus indicating a valid association between SSNHL and vascular etiologies. Second, even though migraine is one of the associated risk factors and in line with the vascular pathophysiology highlighted in this study, its prevalence was not compared between the two cohorts due to limited available information from the control group. Despite these limitations, the exploratory nature of this study can shed light on the partly vascular pathophysiology of SSNHL, which may lead to novel diagnosis and treatment proposals for this poorly understood condition.

## 5. Conclusions

This case-controlled study of 148 SSNHL patients demonstrated the utility of MRI in detecting significantly higher and more progressive WMH in patients with SSNHL compared to controls. By demonstrating higher progressive DWMH and PVH according to Fazekas and Mirsen scales in idiopathic SSNHL, this study suggests a partly vascular pathophysiology for this entity, which further explains its reported associations with other vascular conditions. Future studies to test this hypothesis and develop novel diagnosis and treatment approaches are warranted.

## Figures and Tables

**Table 1:** Average grades of PVHs and DWMHs, assigned using Fazekas and Mirsen scales, among 148 healthy and 150 SSNHL patients.

**Fazekas Grade (Mean ± SD)**			
	**Control**	**SSNHL**	***p*-Value**
PVH	0.020 ± 0.141	1.273 ± 0.684	<0.001
DWMH	0.041 ± 0.198	1.073 ± 0.743	<0.001
**Mirsen Grade (Mean ± SD)**			
	**Control**	**SSNHL**	***p*-Value**
PVH	N/A	N/A	N/A
DWMH	0.372 ± 0.551	2.140 ± 0.859	<0.001

SSNHL = sudden sensorineural hearing loss; PVH = periventricular hyperintensity; DWMH = deep white matter hyperintensity.

**Table 2:** Comparison of PVHs and DWMHs, assigned using Fazekas and Mirsen scales, in 150 healthy and 25 age- and sex-matched SSNHL patients.

**Periventricular Hyperintensities**			
	**Control (%)**	**SSNHL (%)**	***p*-Value**
Fazekas Scale	3/148 (2)	24/25 (96)	<0.001
Mirsen Scale	3/148 (2)	24/25 (96)	<0.001
**Deep White Matter Hyperintensities**			
	**Control (%)**	**SSNHL (%)**	***p*-Value**
Fazekas Scale	6/148 (4)	19/25 (76)	<0.001
Mirsen Scale	51/148 (34)	25/25 (100)	<0.001

SSNHL = sudden sensorineural hearing loss; PVH = periventricular hyperintensity; DWMH = deep white matter hyperintensity.

**Table 3:** Sensitivities and specificities of Fazekas and Mirsen gradings of PVHs and DWMHs from 148 healthy and 150 SSNHL patients.

**Periventricular Hyperintensities**			
	**Fazekas Scale**	**Mirsen Scale**	***p*-Value**
Sensitivity (%)	37/150 (25)	141/150 (94)	<0.001
Specificity (%)	148/148 (100)	145/148 (98)	0.248
**Deep White Matter Hyperintensities**			
	**Fazekas Scale**	**Mirsen Scale**	***p*-Value**
Sensitivity (%)	27/150 (18)	115/150 (77)	<0.001
Specificity (%)	148/148 (100)	143/148 (97)	0.060

SSNHL = sudden sensorineural hearing loss; PVH = periventricular hyperintensity; DWMH = deep white matter hyperintensity.

## Data Availability

The data is available upon reasonable request from the corresponding authors.
